# Therapeutic Targeting of the LXR Pathway for Improving Myelin Integrity in Neurodegeneration

**DOI:** 10.3390/cells15211928

**Published:** 2026-10-23

**Authors:** Sampada Tamhankar, Meenakshi Singh, Joyal Xavier, Kristine Griffett, Rajesh Amin

**Affiliations:** 1 Department of Drug Discovery and Development, Harrison College of Pharmacy, Auburn University, Auburn, AL 36849, USA; spt0019@auburn.edu (S.T.); jzx0049@auburn.edu (J.X.); 2 Department of Anatomy, Physiology and Pharmacology, College of Veterinary Medicine, Auburn University, Auburn, AL 36849, USA

**Keywords:** liver X receptors (LXR), cholesterol homeostasis, 24S-hydroxycholesterol (24S-OHC), CYP46A1, ABCA1, ABCG1, ApoE, oligodendrocytes, myelin, neurodegenerative diseases, demyelinating diseases, neuroinflammation, lipid metabolism, nuclear receptors, drug discovery

## Abstract

**Highlights:**

- LXR targeting offers therapeutic potential for neurodegenerative and demyelinating diseases.
- The CYP46A1/24S-OHC–LXR–ABCA1/ABCG1–ApoE axis regulates CNS lipid homeostasis.
- LXR signaling links brain cholesterol homeostasis to glial and myelin function.
- Next-generation LXRβ-selective and CNS-directed strategies may overcome current therapeutic limitations.

**Abstract:**

Neurodegenerative and demyelinating diseases are often associated with impaired cholesterol homeostasis, glial dysfunction, neuroinflammation, and impaired myelin maintenance. Because myelin is highly enriched in cholesterol and other lipids, efficient lipid transport, recycling, and availability are essential for oligodendrocyte function and remyelination. Liver X receptors (LXRα and LXRβ) are lipid-sensitive nuclear receptors that connect cholesterol metabolism with glial responses and myelin regulation. In the CNS, the 24S-hydroxycholesterol (24S-OHC)-LXR-ABCA1/ABCG1-ApoE axis serves as an important regulatory pathway for cholesterol efflux, ApoE-mediated lipid redistribution, microglial handling of myelin-derived lipids, and maintenance of cholesterol availability required for oligodendrocyte maturation and myelin formation. The 24S-OHC generated from cholesterol by CYP46A1 acts as an endogenous LXR ligand and promotes transcription of cholesterol efflux genes such as ABCA1 and ABCG1, along with ApoE-mediated lipid transport. Although previous studies with LXR agonists have focused on oligodendrocyte health and remyelination via inflammatory regulation, translation of LXR agonists remains limited by poor CNS exposure, lack of isoform selectivity, and LXRα-mediated hepatic lipogenesis and metabolic toxicity. These limitations have shifted therapeutic development toward LXRβ-biased agonists, CNS-restricted delivery, partial agonism, and modulation of the CYP46A1/24S-OHC pathway. Overall, LXR signaling represents a mechanistic link among brain cholesterol homeostasis, glial lipid handling, and myelin integrity; therefore, the development of next-generation selective LXR agonists offers substantial potential to restore lipid homeostasis and enhance neuroprotection across neurodegenerative diseases.

## 1. Introduction

Nuclear receptors (NRs) are a family of ligand-regulated transcription factors that translate metabolic, hormonal, and lipid-derived signals into genetic transcription controlling maturation, metabolism, inflammation, and cellular homeostasis. The human nuclear receptor family consists of 48 members, including peroxisome proliferator-activated receptors (PPARs), retinoid X receptors (RXRs), farnesoid X receptor (FXR), and liver X receptors (LXRs) [1]. These receptors are activated by diverse endogenous ligands, including steroid hormones, thyroid hormone, retinoic acid derivatives, bile acids, fatty acids, and oxysterols [2]. The NR structure consists of a DNA-binding domain (DBD), a ligand-binding domain (LBD), and an activation function (AF) domain, where the gene of interest or DNA binds to the DBD upon ligand activation of the LBD [3]. The binding of the ligand to the receptor triggers a structural change in the receptor, enabling it to recruit cofactors, coactivators, or corepressors, depending on the ligand’s activity as an agonist or antagonist. Additionally, several synthetic ligands can activate the NRs, thereby regulating lipid metabolism, cholesterol transport, glial inflammatory responses, and oligodendrocyte maturation, which are central to myelin maintenance and repair.

The physiological roles of NRs include cellular maintenance, metabolism, development, and inflammation. Multiple nuclear receptors regulate gene transcription by forming heterodimers with retinoid X receptors (RXRs). RXR serves as a heterodimerization partner that facilitates recognition and binding of the receptor complex to specific DNA response elements and contributes to the recruitment of transcriptional coregulators for regulating ligand-dependent regulation of target-gene expression [4]. Based on their binding to RXR, NRs can be classified as permissive or non-permissive heterodimers. Permissive heterodimers such as LXR–RXR, PPAR-RXR, and FXR-RXR are activated when there is a ligand binding to either of the receptors from the primary NR or RXR. Non–permissive heterodimers such as TR-RXR (Thyroid Hormone Receptor), VDR–RXR (Vitamin D Receptor), and RAR-RXR (Retinoic Acid Receptor) strictly need a ligand to bind to the primary NR along with a ligand to RXR to produce effective activation of the receptor [5]. Failure to bind a ligand to the primary NR leads to suppression or inactivity of the receptor and failure of gene transcription associated with that specific receptor. LXR activation requires interaction with RXR to form a heterodimer [6]. Natural endogenous LXR receptor agonists are regulated by cholesterol-derived oxysterols, including 24-hydroxycholesterol, and regulate transcriptional programs involved in cholesterol efflux, lipid redistribution, and inflammatory control. Through transcriptional regulation of target genes, including ABCA1, ABCG1, and ApoE, LXR signaling links cholesterol homeostasis to glial lipid handling and remyelination, making the LXR axis a relevant therapeutic target for myelin loss-related and neurodegenerative diseases [7,8].

## 2. Brain Cholesterol Homeostasis: Origin, Synthesis, Efflux, and the BBB Paradox

The Central Nervous System (CNS) is rich in sterols such as cholesterol, making up roughly one-quarter of the body’s total cholesterol. Most of these sterols are stored in myelin sheaths, with the remainder incorporated into neuronal and glial cell plasma membranes, where they help organize lipid rafts, regulate membrane fluidity, and support receptor function [9,10]. Unlike peripheral circulation, where cholesterol is transported within lipoprotein particles such as ApoB-containing LDL and VLDL and ApoA-I-containing HDL, circulating cholesterol exists in both free (unesterified) and esterified forms, with cholesteryl esters predominantly carried within the hydrophobic core of lipoproteins. In contrast, CNS cholesterol exists primarily in the free, unesterified form, concentrated within myelin sheaths and neuronal membranes, because circulating lipoprotein particles cannot cross the blood–brain barrier (BBB) [11]. Because the BBB becomes impermeable to circulating lipoprotein-bound cholesterol during development, the brain depends almost entirely on local de novo synthesis rather than hepatic or dietary sources.

Within the CNS, cholesterol transport is largely mediated by glial-derived ApoE-containing HDL-like lipoprotein particles rather than peripheral LDL particles, which are excluded by the BBB. Astrocytes are a major source of these particles and redistribute cholesterol to neurons and oligodendrocytes through receptors such as LDLR and LRP1. Cholesterol synthesis is regulated primarily through the SREBP2-HMGCR pathway, whereas excess cellular cholesterol activates LXR-dependent mechanisms that promote cholesterol efflux through ABCA1, ABCG1, and ApoE [9,12]. Together, these pathways maintain a balance between cholesterol synthesis, uptake, redistribution, and removal.

**Figure 1:**
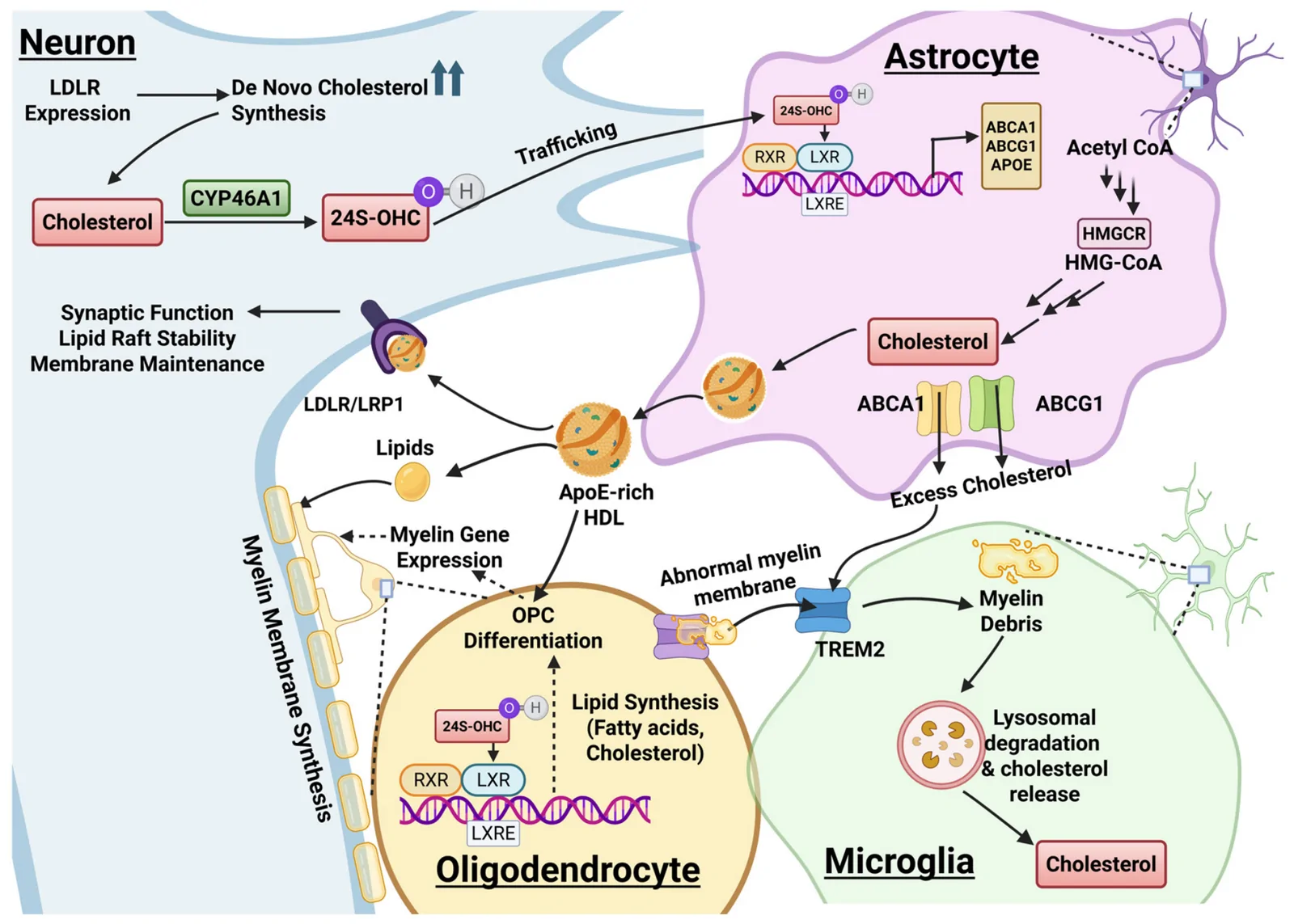
LXR-regulated cholesterol trafficking and glial lipid handling in the CNS. Neuronal cholesterol is converted by CYP46A1 into 24S-hydroxycholesterol (24S-OHC), an endogenous CNS-derived LXR ligand that contributes to brain cholesterol homeostasis and activates LXR signaling. Upon ligand binding, LXR/RXR heterodimers bind to LXR response elements (LXREs) and regulate established lipid homeostasis genes, including ABCA1, ABCG1, and APOE, promoting cholesterol efflux and ApoE-dependent lipid redistribution among CNS cell types. Astrocyte-derived ApoE-containing lipoprotein particles transport lipids to neurons and oligodendrocytes through lipoprotein receptors, supporting cellular cholesterol balance and membrane maintenance. In oligodendrocytes, LXR-mediated regulation of lipid handling promotes cholesterol availability, OPC maturation, and the synthesis of myelin-associated proteins required for myelin sheath formation. Microglia internalize cholesterol-rich myelin debris, process and recycle lipids, and contribute to restoration of a lipid environment supportive of remyelination. Solid arrows indicate established direct regulatory relationships, whereas dashed arrows represent downstream biological effects associated with altered lipid availability, cellular maturation, and myelin repair. The solid black arrows indicate direct downstream signaling, the dotted arrows indicate indirect signaling, and bold (blue) arrows indicate upregulation.

Surplus neuronal cholesterol is mainly converted by CYP46A1 into 24S-hydroxycholesterol (24S-OHC), which can cross the BBB and represents an important pathway for cholesterol elimination from the adult brain. Importantly, 24S-OHC also acts as an endogenous ligand for the LXR-RXR heterodimer. LXR activation promotes expression of cholesterol-handling genes, particularly ABCA1, ABCG1, and ApoE, thereby linking neuronal cholesterol turnover with glial lipid transport and cholesterol redistribution [13] (Figure 1).

Microglia provides an additional route for cholesterol recycling during myelin turnover and injury. TREM2-mediated recognition and uptake of lipid-rich myelin debris is followed by lysosomal degradation, which releases cholesterol for subsequent recycling or efflux [14]. Failure of this lysosomal processing can result in intracellular cholesterol accumulation and impaired lipid handling. Recycled cholesterol can then re-enter ApoE-mediated transport pathways and become available to oligodendrocytes for membrane synthesis and myelin maintenance. In oligodendrocytes, cholesterol obtained through both intrinsic synthesis and ApoE-mediated lipid delivery supports OPC differentiation, myelin gene expression, and expansion of the myelin membrane. Thus, CNS cholesterol homeostasis depends on coordinated interactions among neurons, astrocytes, microglia, and oligodendrocytes rather than on a single cholesterol transport pathway.

Importantly, 24S-OHC is not only an efflux product but also an endogenous LXR ligand. Through this dual role, 24S-OHC links neuronal cholesterol turnover to LXR-dependent transcriptional regulation of cholesterol transport, ApoE lipidation, glial lipid handling, and myelin maintenance. In contrast, other oxysterols, such as 27-hydroxycholesterol, are produced primarily in peripheral tissues by CYP27A1 and can enter the brain from the circulation [15]. Thus, 24S-OHC and 27-OHC represent opposing oxysterol fluxes across the BBB, reflecting the metabolic relationship between brain-intrinsic cholesterol clearance and peripheral cholesterol metabolism.

At the chromatin level, LXR signaling is regulated through ligand-dependent exchange of transcriptional coregulators. In the absence of an activating oxysterol, DNA-bound LXR/RXR heterodimers can associate with the nuclear receptor corepressors NCoR and SMRT, together with HDAC3, thereby maintaining a relatively closed and transcriptionally restrained chromatin state [16]. HDAC3 represents an important epigenetic regulator connecting nuclear receptor signaling with oligodendrocyte development and myelin biology. In the context of LXR signaling, HDAC3 functions primarily as a component of NCoR/SMRT corepressor complexes recruited to unliganded LXR/RXR heterodimers, where it contributes to transcriptional repression of LXR-responsive genes. Following ligand-dependent LXR activation, HDAC3 dissociation permits recruitment of transcriptional coactivators and activation of lipid-homeostasis genes involved in cholesterol transport and metabolism.

Beyond its role in LXR-mediated transcriptional regulation, HDAC3 independently contributes to oligodendrocyte lineage specification and myelin development through chromatin-level regulation of differentiation programs. HDAC3 interacts with p300 and regulates oligodendrocyte-specification pathways involving Olig2-associated transcriptional networks, while restricting alternative astrocytic lineage progression. Loss of HDAC3 disrupts oligodendrocyte lineage commitment and impairs the establishment of mature oligodendrocyte identity, demonstrating its importance in glial fate determination [17].

Furthermore, experimental modulation of HDAC3 activity has demonstrated functional consequences for myelination. Studies in disease-relevant models indicate that altered HDAC3 activity can influence myelin formation, thickness, and functional recovery through regulation of oligodendrocyte differentiation-associated pathways, including PI3K/AKT and MAPK/ERK signaling [18].

These findings suggest that HDAC3 contributes to myelin biology through both transcriptional regulation of oligodendrocyte maturation programs and modulation of signaling pathways required for myelin growth and maintenance. However, whether HDAC3 directly regulates individual structural myelin genes such as MBP, PLP1, MAG, or MOG remains incompletely established. Therefore, HDAC3 should be viewed as an epigenetic regulator that controls oligodendrocyte maturation and myelin-supportive transcriptional programs rather than as a confirmed direct regulator of all structural myelin genes.

**Figure 2:**
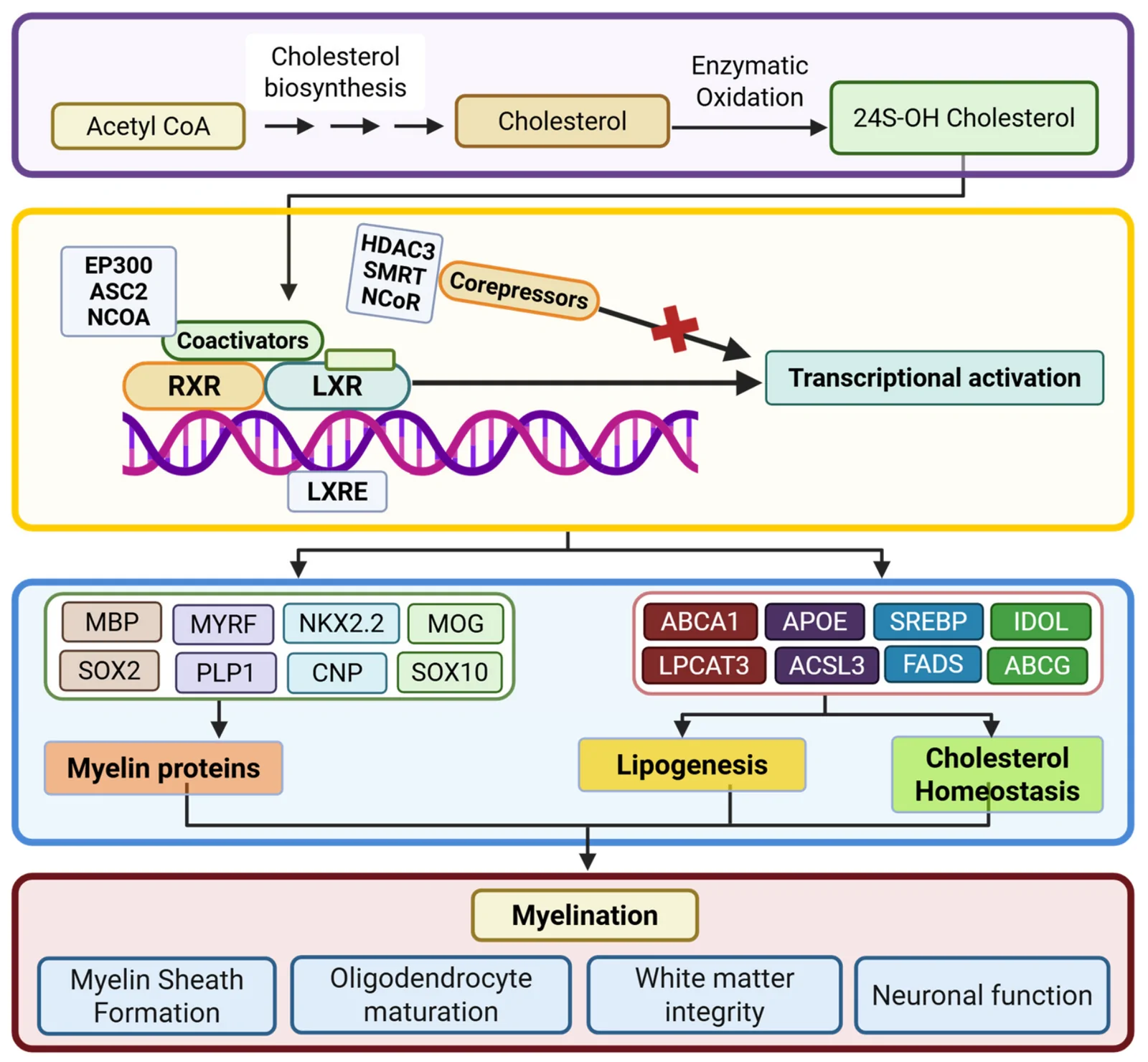
LXR-mediated regulation of cholesterol metabolism and myelination. Acetyl-CoA is metabolized through the cholesterol biosynthesis pathway to generate cholesterol, which can be further oxidized to form 24S-hydroxycholesterol (24S-OHC). This oxysterol serves as an endogenous ligand for liver X receptor (LXR), promoting its activation through heterodimerization with retinoid X receptor (RXR). The activated LXR/RXR complex binds to DNA and regulates transcription of target genes involved in lipid metabolism and myelination. LXR activation upregulates expression of cholesterol transport and lipid metabolism genes, including ABCA1, APOE, and SREBP, leading to enhanced lipogenesis and maintenance of cholesterol homeostasis. In parallel, LXR signaling promotes expression of myelin-associated proteins such as MBP (myelin basic protein) and PLP1 (proteolipid protein 1), supporting myelin protein synthesis. These coordinated processes—lipogenesis, cholesterol homeostasis, and myelin protein production-converge to drive myelination, highlighting the central role of LXR signaling in linking cholesterol metabolism to oligodendrocyte function and myelin integrity. The arrows indicate downstream signaling, and the red cross indicates inhibition.

Oxysterol binding induces a conformational change in LXR that promotes corepressor dissociation and favors recruitment of coactivators such as ASC-2 and p300/CBP, which facilitate histone acetylation, chromatin accessibility, and assembly of the transcriptional machinery [19]. In oligodendrocytes, this regulatory switch couples sterol sensing to the lipid-handling and differentiation programs required for extensive membrane expansion and myelin production. Consistent with this mechanism, loss of LXR signaling impairs oligodendrocyte maturation and decreases the expression of major myelin proteins, including MBP and PLP1, whereas LXR activation promotes myelin-gene promoter activity and enhances remyelination in experimental cerebellar models [20]. Thus, balanced corepressor–coactivator exchange provides a molecular bridge between oxysterol availability, LXR-dependent transcription, and the maintenance of myelin integrity (Figure 2).

Under physiological conditions, this cholesterol cycle maintains a fine balance between synthesis, utilization, and elimination. However, demyelination in neurodegenerative disorders is multifactorial and arises from multiple converging mechanisms, including neuroinflammation, oxidative stress, oligodendrocyte injury, impaired OPC differentiation, defective myelin debris clearance, and altered lipid metabolism. Within this broader pathological context, loss or dysfunction of CYP46A1-expressing neurons represents one cholesterol-related mechanism that can reduce 24S-OHC formation and weaken endogenous LXR activation. In parallel, cholesterol biosynthesis genes, including HMGCR and DHCR24, may be downregulated, contributing to depletion of the intracellular cholesterol pool. Simultaneously, peripherally derived neurotoxic oxysterols like 27-OHC, 7β-hydroxycholesterol, and 7-ketocholesterol accumulate in the brain, driving mitochondrial dysfunction, neuroinflammation, and amyloid-beta production. This shift from a neuroprotective 24S-OHC-dominant state to a neurotoxic 27-OHC/oxidized oxysterol-dominant state represents a core metabolic hallmark of neurodegeneration [21]. When cholesterol clearance and recycling pathways become overwhelmed, excessive and persistent lipid droplet accumulation can occur in glial cells, contributing to microglial activation, inflammatory signaling, and disruption of myelin integrity. Because 24S-OHC functions as an endogenous LXR ligand, reduced 24S-OHC availability may weaken LXR-dependent transcription of cholesterol transport genes such as ABCA1, ABCG1, and ApoE. Restoring or pharmacologically enhancing this pathway could therefore improve cholesterol efflux, ApoE-mediated lipid redistribution, glial lipid handling, and the lipid supply required for oligodendrocyte function and myelin maintenance. Thus, brain cholesterol homeostasis is not a static process but a dynamic axis linking neuronal metabolism, glial function, LXR signaling, and remyelination capacity. This provides the biochemical rationale for investigating the LXR–24S-OHC signaling axis as one potential therapeutic strategy in demyelinating and degenerative diseases.

## 3. Myelin Deficits Across the Neurodegenerative Spectrum

Myelin is highly enriched in cholesterol and other lipids; therefore, disruption of lipid synthesis, cholesterol redistribution, myelin debris clearance, or oligodendrocyte precursor cell (OPC) maturation can compromise white matter integrity. Although myelin is critical for CNS function, its pathological importance differs across neurodegenerative diseases. Myelin loss is a primary pathological feature in multiple sclerosis (MS), where immune-mediated demyelination directly damages oligodendrocytes and axonal myelin. In amyotrophic lateral sclerosis (ALS), myelin dysfunction contributes to motor-neuron vulnerability through impaired oligodendrocyte support, altered MCT1-mediated lactate transport, and disrupted lipid metabolism [22]. In frontotemporal dementia (FTD), C9ORF72, GRN, MAPT, and TDP-43-associated mechanisms contribute to white-matter degeneration and altered myelin architecture [23]. In Alzheimer’s disease (AD), Amyloid-β toxicity, Tau pathology, oxidative stress, and neuroinflammation promote oligodendrocyte dysfunction and secondary myelin loss. Thus, although these disorders differ in their initiating pathology, they converge on impaired oligodendrocyte function, defective myelin maintenance, reduced remyelination capacity, and disrupted axonal conduction, ultimately contributing to network dysfunction, cognitive decline, or motor impairment.

### 3.1. Multiple Sclerosis (MS)

MS is the prototypical immune-mediated demyelinating disease of the CNS [24]. Demyelinating lesions occur in both white and gray matter and commonly involve the optic nerve, spinal cord, periventricular white matter, brainstem, cerebellum, corpus callosum, and cortical/subcortical regions. In these areas, inflammatory infiltration, activated microglia, astrocyte reactivity, T cells, and B cells contribute to oligodendrocyte injury, loss of compact myelin, impaired saltatory conduction, and progressive axonal degeneration. Although oligodendrocyte precursor cells (OPCs) are often present within or around MS lesions, remyelination frequently fails because persistent inflammation, inhibitory myelin debris, extracellular matrix changes, and molecules such as Nogo-A and chondroitin sulfate proteoglycans prevent OPC maturation into functional myelinating oligodendrocytes [25,26]. Current MS therapies primarily reduce immune-mediated injury rather than directly rebuilding myelin [27]. Because myelin debris is cholesterol-rich, defective lipid clearance and altered cholesterol handling may sustain inflammation and impair remyelination. Given the contribution of defective cholesterol handling and impaired OPC maturation to remyelination failure, preclinical demyelination models have increasingly been used to evaluate lipid-directed and nuclear receptor-targeted therapeutic strategies.

Animal model experiments using experimental autoimmune encephalomyelitis (EAE) and Theiler’s Murine Encephalomyelitis Virus (TMEV) models of demyelination have shown increased levels of pro-inflammatory cytokines (e.g., IFN-γ, IL-17, TNF-α) and decreased cholesterol and lipid production, confirming their relevance in studying demyelination pathology [28]. Alternatively, adding 8,9-unsaturated sterols and epoxy/hydroxy cholesterols to these animal models as treatment has demonstrated improvements in myelin maturation markers, suggesting their potential to promote remyelination. These molecules have been combined with drugs targeting Nuclear Receptors (NRs), which also regulate cholesterol homeostasis. NRs such as PPAR and LXR play a critical role in cholesterol regulation and have been shown to transcriptionally regulate OPC maturation by activating myelin-associated genes [29]. Besides NRs, genes involved in cholesterol degradation, such as CYP46A1 and CYP27A1, can also be targeted to promote the formation of specific cholesterol derivatives that enhance myelin formation and support OPC maturation. This underscores a clear need to develop molecules that target lipid homeostasis and OPC differentiation pathways to facilitate remyelination in demyelinating diseases.

### 3.2. Amyotrophic Lateral Sclerosis (ALS)

ALS primarily affects upper and lower motor neurons in the motor cortex, corticospinal tracts, brainstem motor nuclei, and anterior horn of the spinal cord [30]. Although ALS is not classically defined as a primary demyelinating disease, oligodendrocyte dysfunction and myelin abnormalities are increasingly recognized as important contributors to motor-neuron degeneration [31]. In motor pathways, oligodendrocytes provide metabolic support to axons through lactate transport, especially through monocarboxylate transporter 1 (MCT1). Reduced MCT1 function, altered myelin protein expression, oligodendrocyte degeneration, and disrupted cholesterol and sphingolipid metabolism may weaken motor axon support and increase motor-neuron vulnerability. Current ALS therapies do not directly target myelination. Riluzole primarily reduces glutamate-mediated excitotoxicity and provides modest survival benefit [32]. Edaravone acts as an antioxidant and may reduce oxidative stress in selected ALS patients. Tofersen is a mutation-specific antisense oligonucleotide for SOD1-associated ALS and targets mutant SOD1 production rather than myelin repair [33]. These therapies may indirectly protect motor neurons and myelinated motor axons by reducing excitotoxic, oxidative, or genetic injury, but they do not directly promote OPC differentiation, restore oligodendrocyte metabolic coupling, or rebuild myelin in affected motor pathways. This represents an important therapeutic gap. LXR modulation may be relevant because it regulates glial lipid metabolism, ApoE lipidation, cholesterol transport, inflammatory tone, and oligodendrocyte-supportive pathways. However, direct evidence that LXR agonists restore myelination in ALS-affected motor regions remains limited and should be presented as a future research direction rather than an established therapy.

### 3.3. Frontotemporal Dementia (FTD)

FTD is mainly associated with degeneration of frontal and temporal brain networks, but white-matter disruption is increasingly recognized as part of the disease process. Research has shown that there are several causes for the initiation and progression of FTD, including genetic mutations within the genes C9ORF72, MAPT, GRN, VCP, SQSTM1, and UBQLN2 that affect oligodendrocyte biology, PLP1 trafficking, myelin thickness, and axonal g-ratio regulation [34]. Most cases are also characterized by aggregation of Tau and TAR-DNA-binding protein-43 (TDP-43) protein [35]. These alterations may disrupt long-range connectivity through frontal, temporal, callosal, cingulum, and uncinate fasciculus pathways, contributing to behavioral, language, and executive dysfunction.

C9ORF72-associated FTD has been linked to altered myelin thickness and abnormal g-ratio (the ratio of the internal to external diameter of the neuron’s axon) regulation, suggesting impaired axonal myelination. The optimal g-ratio for a healthy neuronal axon is 0.6, which is significantly decreased during FTD, suggesting a reduction in the functional index of optimal axonal myelination, resulting in decreased impulse conduction (13). TMEM106B and GRN-associated mechanisms have also been implicated in oligodendrocyte and myelin abnormalities in FTD [34]. Studies suggest that TMEM106B dysfunction may impair PLP1 trafficking, a process required for compact myelin formation [36].

Current FTD treatment is largely symptomatic. Selective serotonin reuptake inhibitors, trazodone, behavioral therapy, caregiver support, and selected antipsychotic use may help manage behavioral symptoms, agitation, compulsive behavior, or mood-related features. However, these treatments do not directly target oligodendrocyte biology, lipid homeostasis, myelin repair, or remyelination. Disease-modifying therapies for FTD-associated tau, TDP-43, progranulin, or C9ORF72 biology remain under investigation, but no established therapy currently restores myelin integrity in FTD. These findings suggest that altered myelin architecture and oligodendrocyte stress contribute to impaired network connectivity in FTD.

### 3.4. Alzheimer’s Disease (AD)

While AD has traditionally been considered a grey matter disease, increasing neuroimaging, along with histopathological evidence, highlights white matter degeneration, particularly involving oligodendrocyte dysfunction and myelin loss, as a key contributor to disease progression [37]. Recent studies indicate that demyelination in AD is not a sporadic pathology but is driven by specific molecular and cellular mechanisms. Aβ oligomers exert direct toxicity on oligodendrocyte precursor cells (OPCs) and mature oligodendrocytes by inducing apoptotic signaling pathways, including caspase activation and mitochondrial dysfunction. Alongside another central driver of myelin pathology in AD is chronic neuroinflammation, mediated by activated microglia and astrocytes [37]. Activated microglia and astrocytes release inflammatory cytokines such as TNF-α, IL-1β, and IL-6, which impair OPC differentiation and remyelination [38].

Concurrently, Oxidative stress progressively aggravates oligodendrocyte health. Dysregulation of iron and copper homeostasis leads to increased production of reactive oxygen species (ROS), which damage myelin lipids and mitochondrial function in oligodendrocytes [39]. This impairs their ability to provide metabolic support to axons, particularly through lactate transport via monocarboxylate transporters (MCT1). Importantly, demyelination itself contributes to disease progression. Loss of myelin increases axonal energy demand, weakens oligodendrocyte-mediated metabolic support, and exposes axons to further oxidative and inflammatory injury, thereby creating a feed-forward cycle of white-matter and neuronal degeneration. Current AD therapies are not designed to directly promote myelin repair. Cholinesterase inhibitors and memantine mainly target neurotransmission and symptomatic cognitive function. Anti-amyloid monoclonal antibodies, including lecanemab and donanemab, target amyloid burden in early AD and may slow parts of clinical decline, but they do not directly regulate oligodendrocyte maturation or remyelination [40]. Overall, these outcomes show that AD-associated demyelination is driven by amyloid toxicity, neuroinflammatory signaling, oxidative stress, and disrupted glial interactions. Together, these mechanisms position myelin loss as both a consequence and amplifier of AD progression.

## 4. Myelin Loss and Lipid Dysregulation

**Figure 3:**
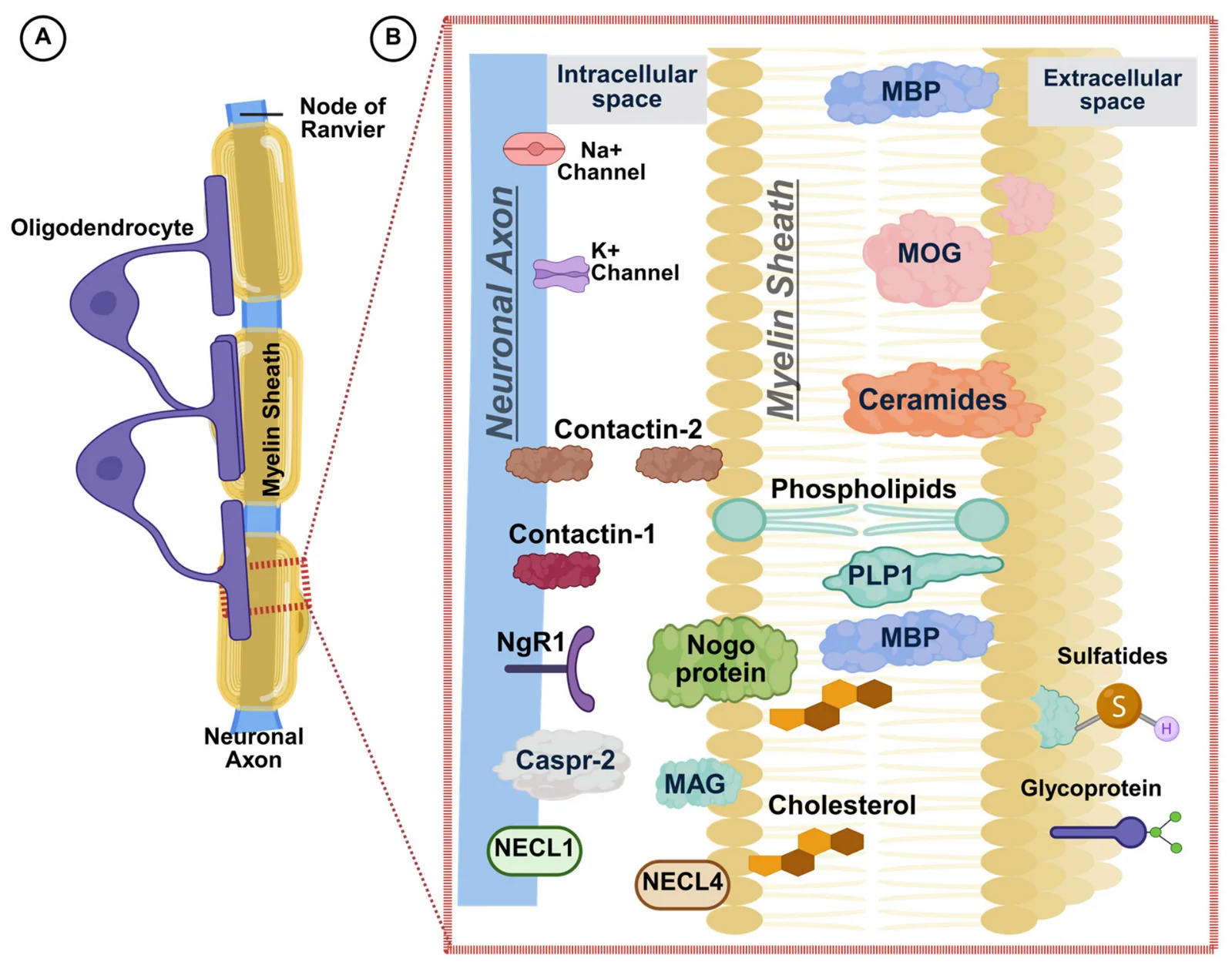
Myelin sheath structure and composition. (**A**). Oligodendrocyte processes wrap around a neuronal axon to form compact multilamellar myelin, separated by nodes of Ranvier. (**B**). Enlarged view of the myelin sheath showing key myelin proteins, including MBP, MOG, PLP1, MAG, and Nogo, along with major lipid components such as cholesterol, phospholipids, ceramides, and sulfatides. Axonal and glial molecules and ion channels support myelin stability and impulse conduction.

Myelin is a specialized multilayer membrane produced by oligodendrocytes in the CNS. Its essential role includes rapid saltatory conduction, axonal stability, metabolic support, and long-term neuronal function. Unlike many cellular membranes, myelin is exceptionally enriched in lipids, particularly cholesterol, sphingolipids, galactocerebrosides, sulfatides, and phospholipids, together with structural proteins such as myelin basic protein (MBP), proteolipid protein 1 (PLP1), myelin-associated glycoprotein (MAG), and myelin oligodendrocyte glycoprotein (MOG) (Figure 3) [41]. This lipid-rich composition gives myelin its compact structure and insulation properties and makes its integrity highly dependent on tightly regulated lipid synthesis, cholesterol recycling, and membrane remodeling.

Myelination is a dynamic developmental and repair process in which oligodendrocyte precursor cells (OPCs) proliferate, migrate, differentiate into pre-myelinating oligodendrocytes, and eventually mature into myelinating oligodendrocytes capable of wrapping axons. This transition requires coordinated activation of transcriptional programs such as OLIG1/2, SOX10, and MYRF, together with strong upregulation of lipid biosynthetic and transport pathways [41]. Mature oligodendrocytes must generate vast amounts of membrane to form myelin sheaths; therefore, cholesterol availability is not merely structural but also regulatory. Insufficient cholesterol supply, defective lipid transport, or impaired lipid raft organization can disrupt oligodendrocyte maturation, reduce expression of myelin proteins, and compromise axonal conduction [42].

Demyelination can arise from multiple converging insults. In MS, immune-mediated attacks on myelin and oligodendrocytes generate inflammatory lesions in which OPCs are often present but fail to differentiate effectively. In AD, amyloid-β toxicity, tau pathology, oxidative stress, and activated microglia and astrocytes contribute to oligodendrocyte dysfunction and white matter degeneration. In FTD, genetic and proteinopathy-related mechanisms involving C9ORF72, GRN, MAPT, TMEM106B, and TDP-43 have been associated with altered myelin thickness, disrupted PLP1 trafficking, and oligodendrocyte stress. In ALS, oligodendrocyte dysfunction, impaired MCT1-mediated lactate support, inflammatory glial activation, and altered cholesterol and sphingolipid metabolism contribute to motor neuron vulnerability. Although these disorders differ in their initiating pathology, they converge on impaired oligodendrocyte support, defective myelin maintenance, and incomplete remyelination [35,43].

A key consequence of demyelination is the release of cholesterol-rich myelin debris into the lesion microenvironment. This debris must be efficiently cleared by microglia and macrophages, processed through lysosomal pathways, and either eliminated or recycled for repair. Following demyelination, lipid droplets can serve as a temporary storage mechanism that allows microglia and macrophages to safely process cholesterol-rich myelin debris. However, when lipid processing, recycling, or export pathways become insufficient, persistent lipid droplet accumulation and cholesterol crystal formation can occur within phagocytes, promoting lysosomal stress, inflammasome activation, and chronic inflammatory signaling. Pro-inflammatory mediators such as TNF-α, IL-1β, and IL-6 can directly inhibit OPC differentiation and suppress remyelination [44]. Thus, demyelination is not only a structural loss of myelin but also a lipid-handling crisis that links cholesterol overload, glial inflammation, and failed repair.

Remyelination requires removal of damaged myelin, regulation of inflammation, OPC differentiation, and sufficient lipid availability for new membrane synthesis. Failure of lipid clearance can lead to cholesterol accumulation within glial cells, prolonged inflammation, and impaired oligodendrocyte maturation [45]. Nuclear receptors are particularly important because they integrate ligand sensing with gene transcription. Several nuclear receptors, including liver X receptors (LXRs), retinoid X receptors (RXRs), peroxisome proliferator-activated receptors (PPARs), and farnesoid X receptors (FXRs), regulate lipid metabolism, cholesterol transport, mitochondrial function, and inflammatory responses [46]. Among these, LXRs are especially relevant to CNS myelin biology because they respond to oxysterols and regulate genes directly involved in cholesterol efflux and lipid redistribution.

Astrocytes are major producers of ApoE-containing lipoproteins and serve as central lipid-distribution hubs in the brain. Through LXR-dependent induction of ApoE, ABCA1, ABCG1, and related lipid-handling genes, astrocytes package cholesterol and phospholipids into HDL-like particles that can be delivered to neurons and oligodendrocytes through receptors such as LDLR and LRP1 [44]. Oligodendrocytes use this lipid supply, together with intrinsic lipid synthesis pathways such as SREBP2-HMGCR-mediated cholesterol biosynthesis, to support membrane expansion, lipid raft organization, and myelin protein expression. Microglia also depend on lipid-handling pathways to engulf myelin debris, recycle cholesterol, and avoid becoming lipid-laden pro-inflammatory cells [47]. Other lipid-sensitive receptors may cooperate with or complement LXR signaling. RXR serves as an obligate heterodimerization partner for LXR and several other nuclear receptors, making RXR signaling an important regulatory node. PPARγ and PPARδ can promote anti-inflammatory glial responses, fatty acid metabolism, and repair-associated transcriptional programs. TREM2, although not a nuclear receptor, is also relevant because it supports microglial recognition of lipid-rich debris and activates DAP12/SYK/PI3K-AKT-mTOR signaling to promote phagocytosis and lipid metabolism [48]. Together, these pathways suggest that remyelination depends on an integrated lipid-regulatory network which is regulated by nuclear receptors.

## 5. Liver X Receptors (LXRs) in the CNS

Liver X receptors (LXRs) are a superfamily of nuclear receptors characterized into two types—LXRα (NR1H3) and LXRβ (NR1H2)—based on their different tissue expression patterns. They are highly expressed in the liver, as well as in the brain, kidney, spleen, small intestine, and adipose tissues [49]. The activation of LXRs by oxysterols was among the first clues indicating their involvement in cholesterol homeostasis [34]. This was confirmed through the analysis of LXRα-knockout mice, which accumulated large amounts of cholesterol in the liver when fed a high-cholesterol diet [49]. Early studies identified the gene encoding cytochrome P450 7A1 (Cyp7a1), the rate-limiting enzyme in bile acid synthesis, as the first direct target of LXRs [50]. A central endogenous activator of CNS LXR signaling is 24(S)-hydroxycholesterol (24S-OHC), also known as 24S-OHC or cerebrosterol. 24S-OHC is produced enzymatically from cholesterol in neurons and acts as a natural ligand for both LXRα and LXRβ [51]. By binding to the LXR ligand-binding domain, 24S-OHC stimulates LXR-dependent transcriptional activity and induces canonical target genes involved in cholesterol efflux, including ABCA1 and ABCG1. In neurons, 24S-OHC binds to LXR and activates genes that help protect the cells and reduce inflammation, providing a safety mechanism for cells in the brain when cholesterol levels rise. Interestingly, 24S-OHC, together with a few other oxysterols like 24,25-epoxycholesterol and 27-OHC, played a key role in showing that LXRs were not “orphan receptors” but have real, biologically relevant ligands [21]. Therefore, 24S-OHC functions not only as a cholesterol metabolite but also as a signaling molecule that couples neuronal cholesterol turnover to transcriptional regulation of lipid handling in the CNS.

LXR activation also exerts anti-inflammatory and anti-oxidative effects that indirectly facilitate OPC differentiation. More specifically, LXR activation suppresses inflammatory cytokines, including TNF-α and IL-1β, from microglia while upregulating genes associated with lipid recycling, thereby reducing the accumulation of myelin debris that otherwise inhibits myelination. Concurrently, LXR signaling intersects with the MYRF–SOX10 axis, two transcription factors essential for initiating myelin gene expression [52]. However, while MYRF and SOX10 directly regulate myelin-associated genes during oligodendrocyte maturation, direct LXRβ binding at the MYRF promoter has not been conclusively demonstrated. Therefore, whether LXR regulates MYRF-dependent myelin gene expression through direct transcriptional mechanisms or indirectly through altered lipid metabolism and cellular maturation remains an important question that requires further investigation.

Following commitment from neural stem cells, OLIG1/2 and SOX10 establish and maintain oligodendroglia identity, whereas proliferative OPCs retain progenitor markers such as PDGFRα, NG2/CSPG4, A2B5, HES5, SOX2, and ID2/4 [48]. At the onset of differentiation, NKX2.2 is transiently upregulated and acts as a molecular gatekeeper controlling the transition from a proliferative OPC to a differentiating oligodendrocyte [53]. NKX2.2 directly represses the PDGFRα promoter, thereby reducing PDGF-dependent mitogenic signaling, OPC proliferation, and migration and permitting activation of the intrinsic differentiation program. Consequently, reduced or dysregulated NKX2.2 activity delays differentiation and allows PDGFRα-driven progenitor programs to persist, as increased NKX2.2 generally favors cell-cycle withdrawal and differentiation [53]. LXRβ expression and several LXR-responsive cholesterol-handling genes increase during oligodendrocyte differentiation, supporting a role for LXR signaling in maintaining the lipid balance required for lineage progression.

As differentiation proceeds, SOX10 directly induces MYRF, after which MYRF and SOX10 cooperatively occupy regulatory regions of genes required for myelin synthesis, including CNP, PLP1, MBP, MOG, and MAG [52]. MYRF simultaneously attenuates OPC-associated SOX10 targets, helping convert the transcriptional program from progenitor maintenance to terminal differentiation and myelination. LXR signaling can support this transition by improving cholesterol efflux and reducing the inflammatory burden associated with myelin debris. LXR-regulated cholesterol handling in lipid-loaded microglia facilitates the removal and recycling of myelin-derived sterols and creates an environment more favorable for remyelination.

**Figure 4:**
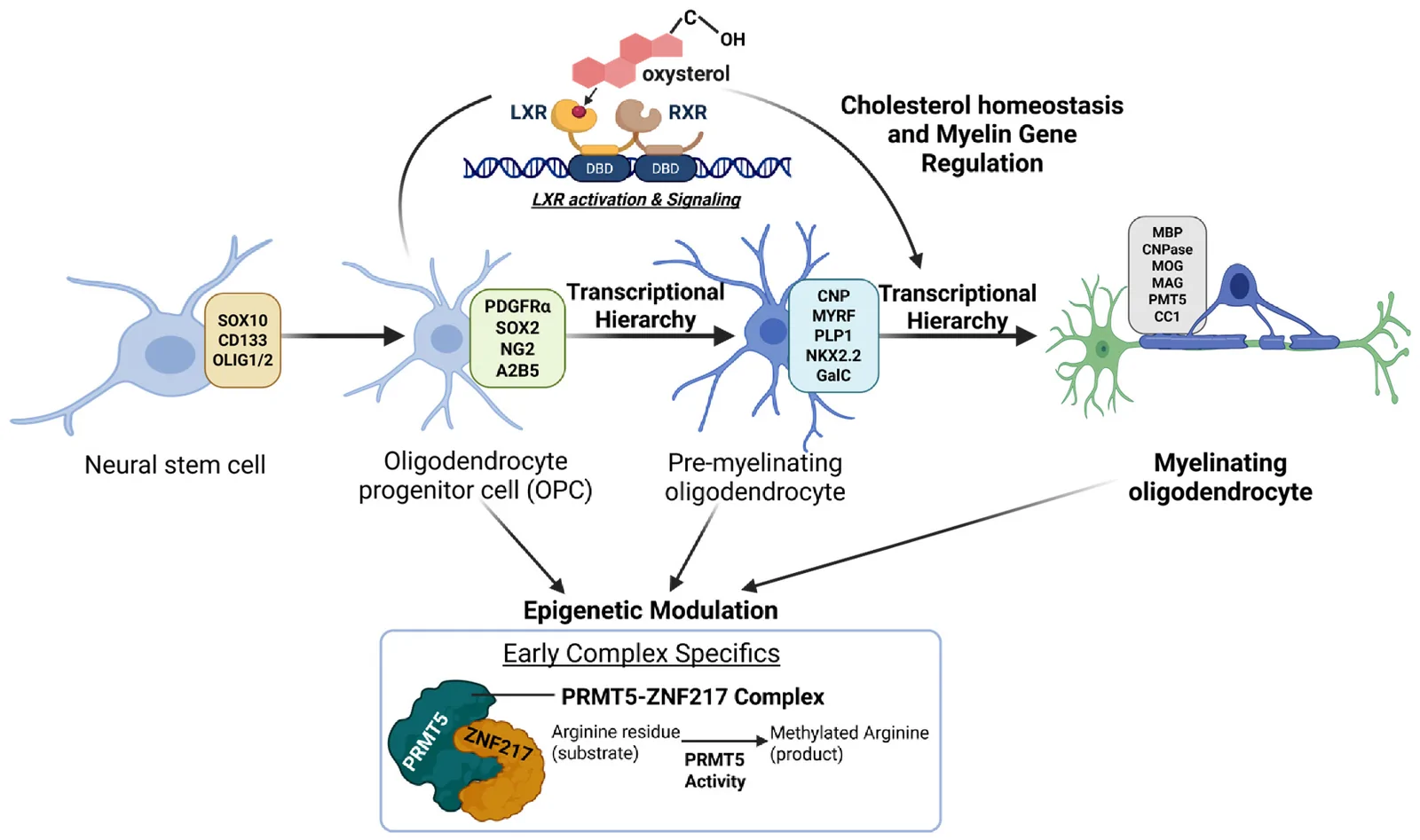
LXR signaling and transcriptional regulation of oligodendrocyte lineage differentiation. Oxysterol-mediated activation of the LXR–RXR heterodimer regulates genes involved in cholesterol homeostasis and myelin formation during oligodendrocyte development. The schematic illustrates progression from neural stem cells to oligodendrocyte precursor cells (OPCs), pre-myelinating oligodendrocytes, and mature myelinating oligodendrocytes, together with representative stage-specific markers. Early differentiation is associated with expression of OLIG1/2, NKX2.2, SOX10, CNP, and related lineage genes, followed by activation of MYRF and mature myelin genes, including MBP, PLP1, MOG, MAG, and CNPase. The transcriptional hierarchy highlights regulation of SOX10 by OLIG2/NKX2.2 and subsequent MYRF-dependent induction of myelin-associated genes. PRMT5-mediated arginine methylation, through its interaction with ZNF217, represents an additional epigenetic mechanism that supports oligodendrocyte differentiation and maturation. The arrows indicate downstream signaling.

An additional epigenetic checkpoint is mediated by protein arginine methyltransferase 5 (PRMT5). PRMT5 is a type II arginine methyltransferase that modifies histone and non-histone substrates through symmetric arginine dimethylation. During the early stages of oligodendrocyte differentiation, PRMT5-mediated methylation supports OPC survival and represses differentiation inhibitors such as ID2 and ID4, thereby permitting progression of the SOX10-MYRF transcriptional program. PRMT5 also influences chromatin organization and the expression of genes required for oligodendrocyte maturation. Loss of PRMT5 maintains an immature transcriptional state, reduces the expression of key differentiation regulators, and impairs the formation of mature myelinating oligodendrocytes. Thus, PRMT5-mediated arginine methylation functions alongside the OLIG2-NKX2.2-SOX10-MYRF hierarchy to establish the transcriptional and epigenetic environment required for stable myelin production and maintenance (Figure 4) [52,53,54,55].

Based on multiple studies, LXR-mediated OPC maturation can be characterized as a three-phase process: Lipid equilibrium phase activation of cholesterol efflux and redistribution pathways; anti-inflammatory clearance phase resolution of inhibitory myelin debris through microglial modulation; and Transcriptional commitment phase-induction of MYRF, SOX10, MBP, and PLP1 for myelin assembly [7,56]. Experimental loss of LXR signaling results in reduced myelin-gene expression and thinner myelin, whereas pharmacological LXR activation promotes oligodendrocyte maturation and remyelination [54]. Thus, LXR can be positioned across the myelination process as a regulator of cholesterol homeostasis during OPC development, a facilitator of the lipid and inflammatory environment required for differentiation, and a supporter of lipid availability during mature myelin formation.

## 6. LXR and Its Role in Myelination

Liver X Receptors exist in two closely related isoforms—LXRα (NR1H3) and LXRβ (NR1H2)—which share high sequence homology yet differ markedly in tissue distribution and physiological function [57]. This distinction has profound implications for therapeutic design: while both isoforms respond to oxysterols such as 24S-OHC, activation of hepatic LXRα can trigger undesirable lipogenesis and hypertriglyceridemia, whereas targeting LXRβ offers neuro-selective benefits with fewer systemic liabilities.

Within the CNS, LXRβ plays a central role in lipid trafficking required for myelination. Genetic knockout studies demonstrate that mice lacking LXRβ exhibit hypomyelination, reduced expression of myelin basic protein (MBP) and proteolipid protein (PLP1), and delayed remyelination following injury [58]. These findings emphasize that LXRβ functions as the primary LXR isoform responsible for oligodendrocyte differentiation and myelin maintenance. Conversely, systemic activation of LXRα induces hepatic lipogenic genes such as SREBP-1c, leading to fatty-liver phenotypes that have hampered clinical translation of early LXR agonists (e.g., T0901317, GW3965) [59].

Therefore, isoform selectivity represents both a mechanistic insight and a pharmacological necessity. Future LXR-targeted therapeutics aim to target the neuroprotective and pro-myelinating actions of LXRβ while sparing peripheral LXRα-driven pathways. This can be achieved through CNS-penetrant, LXRβ-biased agonists or tissue-restricted delivery systems such as intranasal administration. Understanding these distinctions underscores why LXRβ selectivity is a critical target while designing next-generation remyelination therapies.

Furthermore, the pharmacodynamics of LXR activation can span from partial to full agonism, each conferring distinct transcriptional profiles. Full agonists such as T0901317 activate receptors to the maximum across both isoforms—LXRα and LXRβ—strongly inducing cholesterol efflux genes but also lipogenic genes such as SREBP-1c and FASN, leading to excessive hepatic lipid synthesis [60]. In contrast, partial agonists engage the receptor in a conformation that selectively recruits co-activators that target the activation of neuroprotective and pro-myelinating genes, while minimizing lipogenic responses.

Recent medicinal-chemistry efforts have identified compounds such as N, N-dimethyl-3β-hydroxycholenamide (DMHCA) and LXR-623 that exhibit partial agonism or biased signaling toward LXRβ [61,62]. These compounds preserve induction of CNS-relevant targets such as ABCA1 and APOE while reducing hepatic triglyceride accumulation compared with nonselective full agonists. Similarly, dual modulators such as AU403 represent an emerging strategy to fine-tune LXR activity by balancing neuroprotective and metabolic effects. Structurally, T0901317 forms a key interaction with His435 in LXRβ and His421 in LXRα near helix 12, whereas GW3965 adopts an alternative orientation within the pocket. Both ligands stabilize the C-terminal helix 12/AF-2 region in an active conformation that promotes coactivator recruitment and transcriptional activation [63,64]. In contrast, AU403 preferentially interacts with residues within the LXRβ pocket, including Phe329 and Leu330, while avoiding corresponding LXRα interactions, providing a structural basis for its LXRβ preference and altered pharmacological profile. Thus, ligand-specific contacts within the LBD can influence the conformation of helices 3, 11, and particularly helix 12/AF-2, thereby altering cofactor recruitment and downstream transcriptional activity [65,66]. Rather than maximizing receptor activation, these approaches aim to achieve a transcriptional profile that supports cholesterol redistribution, oligodendrocyte function, and remyelination while minimizing systemic metabolic toxicity.

From a therapeutic standpoint, partial agonism represents a critical balance between efficacy and safety. By fine-tuning receptor activation, researchers can exploit the beneficial effects of LXRβ—cholesterol redistribution, anti-inflammatory signaling, and OPC maturation—while avoiding the detrimental metabolic consequences of LXRα overactivation. This newer pharmacological control is increasingly recognized as the most promising route for developing next-generation LXR modulators tailored to promote myelination and neuroprotection in chronic CNS disorders.

### 6.1. Mechanisms of LXR-Mediated Myelin Repair

LXR-mediated myelin repair involves several interconnected mechanisms that regulate cholesterol recycling, oligodendrocyte precursor cell (OPC) maturation, myelin gene expression, microglial debris clearance, and inflammatory resolution. Because myelin is highly enriched in cholesterol and other lipids, effective remyelination requires not only the generation of new oligodendrocytes but also the coordinated clearance, redistribution, and reuse of cholesterol within the CNS.

#### 6.1.1. Cholesterol Efflux and Lipid Recycling

Demyelination in the CNS can result from oligodendrocyte death or injury, and failures in cholesterol clearance often exacerbate it [67]. NPC1 and NPC2 support lysosomal cholesterol trafficking, allowing internalized cholesterol to move out of lysosomes for reuse or export [68]. Cholesterol turnover is further regulated by CYP46A1 and CH25H, which convert cholesterol into oxysterols that facilitate cholesterol elimination and can also contribute to LXR activation. LDLR supports uptake of ApoE-containing lipoproteins and redistribution of cholesterol between CNS cells [69,70]. At the same time, remyelination requires sufficient cholesterol for new membrane synthesis. SREBF2 regulates cholesterol biosynthesis through downstream genes including HMGCR, HMGCS1, and DHCR24, which help maintain the lipid supply required for oligodendrocyte maturation and myelin formation [71]. RXR, the heterodimeric partner of LXR, is also required for effective LXR-dependent transcription. In the absence of activating ligands, LXR/RXR complexes remain bound to LXR response elements (LXREs) while recruiting corepressor complexes, including NCoR, SMRT, and HDACs, resulting in transcriptional repression. The binding of endogenous oxysterols, such as 24S-hydroxycholesterol (24S-OHC), or synthetic agonists induces a conformational change that promotes corepressor release and recruitment of coactivators, including SRC family proteins and CBP/p300, enabling transcription of LXR-responsive genes. Canonical direct LXR targets, including ABCA1, ABCG1, and APOE, regulate cholesterol efflux, lipid transport, and cellular lipid homeostasis. In contrast, increased expression of myelin-associated proteins such as MBP, PLP1, MAG, and MOG following LXR activation is currently considered a downstream consequence of improved lipid availability, oligodendrocyte maturation, and reduced inflammatory signaling primarily, as direct LXR binding at these loci remains incompletely established.

Thus, successful remyelination depends on coordinated regulation of cholesterol uptake, lysosomal processing, efflux, turnover, and synthesis rather than simply increasing or decreasing total cholesterol levels. Hence, LXR activation supports both the removal of excess cholesterol from damaged tissue and the recycling of lipids required for new myelin membrane synthesis. In this way, the 24S-OHC-LXR-ABCA1/ABCG1-ApoE axis links neuronal cholesterol turnover, microglial debris clearance, glial lipid transport, and myelin maintenance.

#### 6.1.2. OPC Differentiation

Oligodendrocyte precursor cells (OPCs) undergo a multistage transition from progenitor cells to pre-myelinating cells and, finally, to mature oligodendrocytes. This transition requires strong upregulation of lipid biosynthesis because mature oligodendrocytes must generate extensive myelin membrane. Myelin sheaths are composed of roughly 70% lipids by dry weight, dominated by cholesterol, galactocerebrosides, and sphingomyelin [72,73]. During differentiation, cholesterol functions not only as a structural component but also as a regulator of membrane curvature, cytoskeletal remodeling, and cellular maturation.

Insufficient cholesterol availability halts OPC maturation, leading to hypomyelination and impaired axonal conductivity. LXR signaling supports OPC differentiation by improving cholesterol efflux, lipid redistribution, and intracellular lipid balance. Experimental induction of LXRβ using 24S-OHC or synthetic agonists promotes this transcriptional switch by enhancing cholesterol efflux and redistribution, thereby supplying membrane lipids for myelin formation [54].

#### 6.1.3. LXR-Regulated Transcriptional Programs Supporting Myelin Formation

LXR-mediated regulation of myelin formation occurs primarily through control of the lipid environment required for oligodendrocyte maturation and membrane biogenesis. Although canonical LXR targets such as ABCA1, ABCG1, and APOE are directly regulated through LXR response elements (LXREs), the relationship between LXR activation and structural myelin genes requires careful distinction. Current evidence supports that LXR activation promotes myelin-associated gene expression through a combination of direct lipid-regulatory transcription and indirect effects on oligodendrocyte maturation, cholesterol availability, and inflammatory resolution.

During oligodendrocyte differentiation, transcription factors including OLIG2, NKX2.2, SOX10, and MYRF coordinate the transition from OPCs into mature myelinating oligodendrocytes. This process requires substantial lipid synthesis because oligodendrocytes must generate extensive cholesterol-rich membrane structures to form the myelin sheath. By increasing expression of cholesterol transport and lipid-handling genes, LXR activation enhances cholesterol redistribution and provides the lipid substrates required for membrane expansion. This improved lipid environment supports activation of the oligodendrocyte maturation process and increased expression of myelin-associated proteins, including MBP, PLP1, MAG, MOG, and CNP [41].

However, whether individual structural myelin genes represent direct LXR transcriptional targets remains incompletely established. Unlike ABCA1, ABCG1, and APOE, which contain characterized LXR-responsive regulatory regions, promoter-level evidence demonstrating direct LXR binding at MBP, PLP1, MAG, or MOG loci is limited. Therefore, increased myelin protein expression following LXR activation should currently be interpreted as a downstream consequence of improved cholesterol availability, lipid redistribution, enhanced OPC maturation, and reduced inflammatory inhibition rather than confirmed direct transcriptional activation.

Future studies using LXRE mapping, LXRα/LXRβ ChIP-seq or CUT&RUN, promoter-reporter assays with LXRE mutations, and oligodendrocyte-specific transcriptomic approaches will be necessary to determine which myelin-associated genes are directly regulated by LXR signaling. Establishing this distinction will be critical for defining whether LXR functions primarily as a master regulator of the lipid environment required for myelination or as a direct transcriptional regulator of myelin structural genes.

#### 6.1.4. ApoE-Dependent Lipid Handling in Myelin Maintenance and Repair

**Figure 5:**
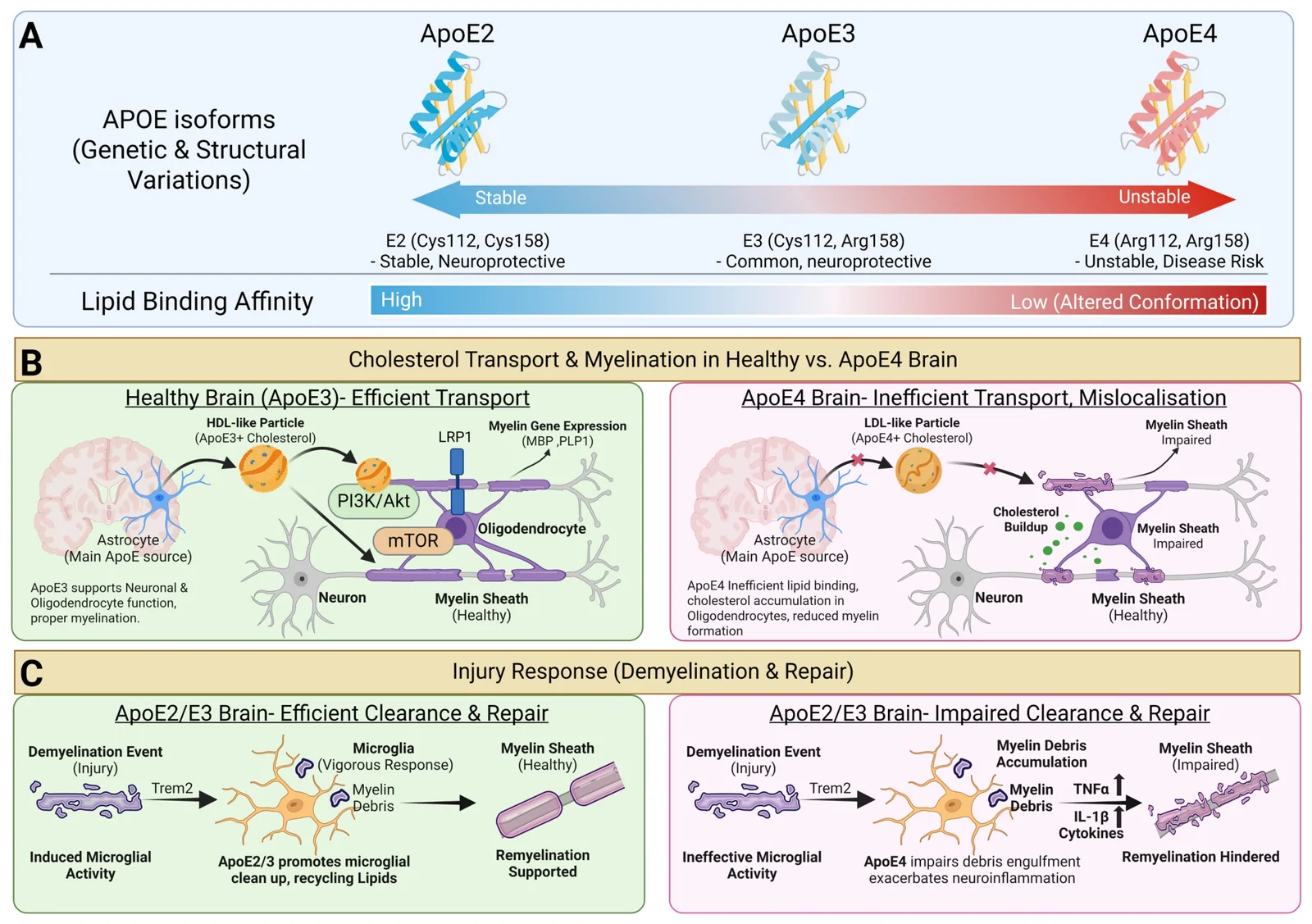
ApoE isoform–dependent regulation of lipid transport, myelination, and injury response in the brain. (**A**) Structural and functional differences among ApoE isoforms. ApoE2, ApoE3, and ApoE4 differ by amino acid substitutions at positions 112 and 158, influencing structural stability, lipid-binding capacity, and disease risk. (**B**) ApoE isoforms differentially regulate cholesterol transport and myelination. In the healthy brain (ApoE3), astrocyte-derived ApoE-containing HDL-like particles efficiently deliver cholesterol to neurons and oligodendrocytes via receptors such as LRP1. This promotes activation of PI3K/AKT and mTOR signaling pathways, enhancing expression of myelin-associated genes (e.g., MBP, PLP1) and supporting proper myelin sheath formation. In contrast, the ApoE4 brain demonstrates impaired lipid transport and mislocalization of cholesterol. ApoE4-associated particles exhibit reduced efficiency in cholesterol delivery, leading to lipid accumulation, disrupted oligodendrocyte function, and compromised myelin sheath integrity. (**C**) ApoE isoforms influence injury response, demyelination, and repair. Following demyelinating injury, ApoE2/E3 facilitates an effective repair response by promoting microglial activation (e.g., via TREM2), efficient clearance of myelin debris, and lipid recycling, thereby supporting remyelination. In contrast, ApoE4 impairs microglial function, resulting in defective debris clearance, accumulation of myelin fragments, and heightened neuroinflammation characterized by increased cytokine production (e.g., TNF-α, IL-1β). These processes collectively hinder remyelination and exacerbate myelin damage. The arrows indicate downstream signaling, and the red crosses indicates inhibition.

Efficient myelin maintenance and remyelination depend on coordinated lipid handling and signaling between astrocytes, microglia, and oligodendrocytes. Apolipoprotein E (ApoE) is the dominant apolipoprotein in CNS lipoproteins and plays an important role in redistributing lipids among glial cells and neurons during injury repair [74]. During demyelination, microglia must engulf cholesterol-rich myelin debris, process the lipid burden, and recycle cholesterol in a manner that supports tissue repair rather than chronic inflammation. In the healthy CNS, Astrocytes synthesize cholesterol and package it into ApoE-containing HDL-like lipoprotein particles, which are released into the extracellular space [75]. These ApoE-lipid particles transport cholesterol to neurons and oligodendrocytes through lipoprotein receptors such as LDLR and LRP1 (Figure 5B). Oligodendrocytes then use the delivered cholesterol for membrane synthesis and formation and maintenance of the cholesterol-rich myelin sheath. Thus, ApoE functions as a major lipid carrier that connects astrocytic cholesterol production with oligodendrocyte requirements.

ApoE isoforms can alter the efficiency of this lipid transport. ApoE2 and ApoE3 generally support more effective cholesterol transport and redistribution, whereas ApoE4 is associated with impaired lipid trafficking and greater intracellular lipid accumulation [76] (Figure 5A). Reduced cholesterol delivery or abnormal lipid redistribution can limit the lipid supply available for oligodendrocyte function and myelin maintenance. Lipoprotein-receptor signaling, including LRP1-associated PI3K/AKT and mTOR pathways, may also contribute to oligodendrocyte survival, differentiation, and myelin production [77]. Following demyelination, the direction of lipid handling changes. Microglia engulf cholesterol-rich myelin debris and process the accumulated lipids (Figure 5C). Cholesterol recovered from degraded myelin can then be exported via pathways involving ABCA1, ABCG1, and ApoE, thereby redistributing lipids rather than retaining them within microglia. Efficient recycling helps prevent lipid-droplet accumulation and supports a lesion environment that is more favorable for oligodendrocyte differentiation and remyelination. In contrast, ApoE4-associated defects in lipid transport and microglial cholesterol handling can promote lipid accumulation, persistent inflammation, and impaired myelin repair [78,79].

The therapeutic efficacy of Liver X Receptor (LXR) activation within demyelinating and neurodegenerative microenvironments is profoundly gated by APOE polymorphism, revealing a distinct cell-type-specific paradox in APOEε4 carriers that differs between LXRα and LXRβ signaling networks. In microglia, ApoE4 drives functional stagnation by disrupting the physical interaction between the epigenetic co-activator Asxl1 and LXRα. This loss of interaction depletes active H3K4me3 histone marks at the promoter regions of critical lipid-efflux targets, effectively silencing baseline transcription of ABCA1 and ABCG1, and trapping microglia in a chronic, lipid-laden “foamy” phenotype marked by defective TREM2-mediated myelin clearance. Small-molecule targeting of LXRβ-the dominant LXR isotype in the central nervous system presents a vital pharmacological bypass. Exogenous LXRβ activation successfully overrides this localized LXRα-specific epigenetic blockade to restore microglial ABCA1 expression, evacuate toxic intracellular cholesterol esters, and blunt baseline pro-inflammatory signaling (TNFα, IL-1β) across all genotypes [80,81].

However, evaluating the system through an LXRβ lens simultaneously reveals why APOEε4 tissue exhibits profound relative resistance to downstream remyelination. Beyond its role in microglia, LXRβ acts as a direct cell-intrinsic regulator of oligodendrocyte progenitor cell (OPC) differentiation and structural myelin protein synthesis (such as MBP and PLP). In a permissive APOEε2 or APOEε3 environment, LXRβ agonism successfully coordinates the extracellular provision of lipid building blocks with internal myelin sheath assembly. Conversely, in the APOEε4 background, this dual-action program is uncoupled: despite LXRβ-driven transcriptional activation of the myelin program, APOEε4 oligodendrocytes suffer from an intrinsic loss of function where imported or newly synthesized cholesterol is mislocalized and trapped within endolysosomal compartments. This intracellular sequestration starves the expanding plasma membrane of structural lipids, stalling OPC maturation and rendering the APOEε4 niche functionally resistant to LXR-mediated remyelination despite successful microglial lipid clearing.

#### 6.1.5. Anti-Inflammatory Signaling

LXR-mediated myelin repair also depends on limiting chronic inflammation. During myelin repair, lipid droplets may initially support microglial adaptation by providing temporary storage for cholesterol-rich myelin-derived lipids. However, in the setting of impaired lipid processing, excessive lipid droplet retention and cholesterol crystallization can trigger lysosomal damage and inflammasome activation, driving microglia toward a persistent pro-inflammatory phenotype. In demyelinating diseases such as MS, aged or lipid-overloaded microglia can become “foamy” and less capable of clearing myelin debris, leading to sustained inflammation and impaired remyelination [82]. Similarly, neurodegenerative diseases such as AD and FTD, chronic inflammation and disrupted cholesterol homeostasis in glia are associated with white matter degeneration.

Inflammatory cytokines further impair repair. Inefficient debris clearance, particularly in ApoE4-associated contexts, can elevate TNFα and IL-1β, which inhibit OPC differentiation and hinder remyelination. LXR activation may counter this process by promoting ABCA1/ABCG1-mediated cholesterol efflux, ApoE-dependent lipid redistribution, and anti-inflammatory transcriptional responses. By reducing lipid overload and inflammatory signaling, LXR activation helps shift the CNS microenvironment from a degenerative state toward a pro-remyelinating state that supports OPC maturation, myelin gene expression, and myelin repair [45]. Together, these mechanisms show that LXR-mediated myelin repair is not limited to cholesterol efflux alone. Instead, LXR activation coordinates lipid recycling, oligodendrocyte maturation, myelin gene expression, microglial debris clearance, and inflammatory resolution, making it a central pathway linking cholesterol homeostasis to remyelination capacity in neurodegenerative and demyelinating diseases.

## 7. Drug Discovery Landscape and Current Therapeutics and Limitations

Current treatments for neurodegenerative and demyelinating diseases provide important clinical benefits but generally do not directly restore myelin integrity. In Alzheimer’s disease, approved therapies such as cholinesterase inhibitors, memantine, and anti-amyloid monoclonal antibodies primarily target neurotransmission or amyloid burden rather than oligodendrocyte function or remyelination [83,84,85,86]. In ALS, Riluzole and Edaravone provide modest disease-modifying or antioxidant benefit but do not directly repair oligodendrocyte dysfunction or myelin loss [32,33]. In FTD, therapeutic options remain largely symptomatic, as currently there is no FDA-approved treatment for FTD [87]. In MS, disease-modifying therapies reduce immune-mediated inflammatory activity and relapse frequency, but remyelination failure and progressive neurodegeneration remain major unmet needs [26,88,89]. Therefore, across these disorders, a key therapeutic gap is the lack of strategies that directly target lipid homeostasis, glial cholesterol handling, OPC maturation, and myelin repair.

LXR signaling has emerged as a novel signaling pathway to address this gap because it regulates several processes central to myelin maintenance, including cholesterol efflux, ApoE-mediated lipid transport, microglial lipid clearance, inflammatory resolution, and oligodendrocyte maturation. Unlike therapies that target a single disease-specific pathology, LXR modulation acts on a shared lipid-regulatory axis that is relevant across multiple neurodegenerative and demyelinating conditions. Activation of the 24S-OHC-LXR-ABCA1/ABCG1-ApoE pathway, together with other LXR-centered and myelin-relevant therapeutic strategies summarized in Table 1, may improve cholesterol redistribution, enhance myelin debris clearance, reduce inflammatory glial activation, and support the lipid supply required for oligodendrocyte membrane expansion during remyelination.

Early synthetic LXR agonists, including T0901317 and GW3965, have provided proof-of-concept evidence that pharmacological LXR activation can enhance cholesterol efflux, increase ApoE lipidation, reduce inflammatory signaling, and support remyelination-associated outcomes in experimental models [8,55,93]. However, these compounds are non-selective pan-LXR agonists and activate both LXRα and LXRβ. This is therapeutically important because LXRβ is more relevant to CNS glial and oligodendrocyte biology, whereas systemic LXRα activation is associated with hepatic lipogenesis, hypertriglyceridemia, and fatty liver [94]. Therefore, the drug discovery field has shifted from broad LXR activation toward more selective strategies, including LXRβ-biased ligands, CNS-restricted agonists, intranasal delivery, nanoparticle-based formulations, prodrugs, and indirect activation through CYP46A1/24S-OHC modulation.

Overall, the drug discovery landscape suggests that LXR modulation is most promising when approached as a targeted CNS lipid-repair strategy rather than as broad systemic LXR activation. Future therapeutic development should prioritize compounds or delivery systems that enhance LXRβ-driven cholesterol handling in the CNS, increase ApoE lipidation and ABCA1/ABCG1-mediated efflux, promote microglial clearance of cholesterol-rich myelin debris, and support oligodendrocyte maturation. Given the central role of 24S-OHC–LXR signaling in coupling cholesterol turnover to anti-inflammatory and pro-myelinating gene programs, this pathway is a compelling therapeutic target for demyelinating and neurodegenerative diseases. As illustrated in Figure 2, LXR activation coordinates a multi-step cascade: upstream cholesterol metabolism (including oxidation to oxysterols such as 25-OH cholesterol) drives RXR-LXR heterodimerization at LXR response elements (LXREs), leading to transcriptional activation of myelin structural proteins (MBP, PLP1, CNP, MOG) and lipid-regulatory genes (ABCA1, APOE, SREBP) whose integrated outputs ultimately govern myelination, oligodendrocyte maturation, white matter integrity, and neuronal function.

LXR agonists (synthetic or natural) have shown promise in experimental models. Activation of LXR in demyelinated CNS tissue upregulates ApoE, ABCA1, and ABCG1, facilitating cholesterol clearance, and it shifts microglia/macrophages to a repair-friendly phenotype [10]. Interestingly, treating demyelinated mice with LXR agonists reduces the number of foamy, lipid-containing microglia and accelerates lesion resolution and remyelination. LXR activation also directly enhances oligodendrocyte differentiation and myelin gene expression: studies show that 24S-OHC or synthetic LXR ligands can increase levels of myelin basic protein (MBP) and proteolipid protein (PLP) in oligodendrocytes, leading to thicker myelin sheaths and faster functional recovery in models of injury [54]. For example, mice lacking LXRα/β have thinner myelin and delayed remyelination, whereas pharmacological LXR stimulation promotes new myelin formation after a demyelinating process [58].

Despite strong mechanistic rationale and promising preclinical findings, several limitations have restricted the therapeutic translation of LXR-targeted strategies for neurodegenerative and demyelinating diseases. A major challenge is the lack of isoform selectivity among early synthetic LXR agonists. Full agonists such as T0901317 and GW3965 activate both LXRα and LXRβ. Systemic LXR agonists can directly activate LXRα in the liver, inducing lipogenesis and adverse effects like hypertriglyceridemia, fatty liver, and even peripheral immunosuppression, such as neutropenia [95,96]. These side effects have affected clinical trials of many clinical-stage LXR agonists. Although this can enhance cholesterol efflux and ApoE-mediated lipid transport, systemic activation of hepatic LXRα induces lipogenic pathways, including SREBP-1c-dependent fatty acid synthesis, leading to hypertriglyceridemia, hepatic steatosis, and other metabolic liabilities. These adverse effects limit chronic dosing and remain a central barrier for clinical development. To overcome this, current strategies include developing brain-selective or LXRβ-specific agonists (since LXRβ is the predominant isoform in CNS glia) that spare LXRα in peripheral tissues. Another approach is targeted delivery—for instance, intranasal administration of an LXR agonist has been shown to raise CNS ApoE levels and improve cognition in an AD model without causing hyperlipidemia [97].

A second limitation is inefficient CNS delivery. Many LXR agonists have poor brain penetration because of unfavorable physicochemical properties, peripheral metabolism, or limited blood-brain barrier permeability [98]. As a result, systemic administration may produce stronger peripheral effects than CNS-specific therapeutic benefit. Future drug development should therefore prioritize CNS-penetrant or CNS-restricted approaches that increase brain exposure while minimizing hepatic activation. Potential strategies include intranasal delivery, nanoparticle-based formulations, prodrugs, and tissue-restricted agonists designed to preferentially activate LXR signaling within the CNS.

Isoform selectivity is another critical direction. Since LXRβ is highly expressed in CNS cell types, including neurons, astrocytes, microglia, and oligodendrocytes, whereas LXRα is more strongly associated with hepatic lipid metabolism, LXRβ-biased agonists may offer a more favorable therapeutic window [99].

Another promising approach is indirect modulation of the LXR pathway through endogenous oxysterol metabolism. Because 24S-OHC functions as a brain-derived endogenous LXR ligand, strategies that restore CYP46A1 activity or normalize 24S-OHC production may enhance LXR signaling in a more physiologically restricted manner. This approach could potentially strengthen neuronal cholesterol clearance, improve glial lipid handling, and support myelin maintenance without the same degree of systemic LXRα activation observed with nonselective synthetic agonists. However, this strategy requires careful validation because excessive or imbalanced oxysterol signaling may also contribute to oxidative stress and inflammation. Therefore, CYP46A1-based approaches should be evaluated not only by measuring 24S-OHC elevation but also by assessing oxysterol balance, including the 24S-OHC/27-OHC ratio, accumulation of potentially toxic oxysterols such as 7-ketocholesterol, oxidative stress markers including lipid peroxidation and reactive oxygen species, mitochondrial function, and cellular viability. A successful therapeutic strategy would be expected to restore physiological cholesterol turnover, enhance LXR-dependent lipid transport programs (ABCA1, ABCG1, and ApoE), and promote oligodendrocyte function, while avoiding excessive accumulation of oxidized sterols and metabolic disruption.

A further limitation is the need for better disease-relevant models. Much of the current evidence for LXR-mediated remyelination comes from cell culture systems, acute demyelination models, or immune-mediated models such as EAE. While these models are valuable, they do not fully reproduce the chronic, heterogeneous, and age-associated myelin pathology seen in neurodegenerative diseases such as AD, FTD, ALS, and progressive MS. Although cuprizone, EAE, and TMEV models have provided important insights into LXR-dependent lipid regulation and remyelination, these models primarily represent acute demyelination or inflammatory injury and may not fully reproduce the progressive myelin dysfunction observed in aging-associated neurodegenerative diseases.

Future studies should therefore incorporate aged animals, chronic demyelination paradigms, and disease-specific genetic models to determine whether LXR modulation can preserve oligodendrocyte function and promote remyelination under disease-relevant conditions. AD models such as APP/PS1 and 5xFAD, tauopathy models associated with FTD, and ALS models involving SOD1, TDP-43, C9ORF72, or FUS mutations may provide valuable platforms for evaluating long-term effects of LXR activation on white matter integrity and neuronal support. In addition, human iPSC-derived neuron–oligodendrocyte–microglia co-cultures and cerebral organoid systems will be particularly important for assessing human-specific responses and interactions between lipid metabolism, inflammatory signaling, and myelin formation.

These models should include direct readouts of OPC maturation, myelin protein expression, lipidomic remodeling, ApoE lipidation, 24S-OHC/27-OHC balance, microglial lipid burden, and functional remyelination. Evaluation of therapeutic efficacy should incorporate structural, molecular, metabolic, and functional endpoints, including electron microscopy-based g-ratio analysis, myelin thickness measurements, MBP/PLP1/MAG/MOG expression, oxysterol quantification, OPC differentiation, and MCT1-mediated metabolic coupling between oligodendrocytes and neurons. Together, these integrated approaches will help distinguish true functional remyelination from isolated changes in molecular markers and provide a clearer path toward clinical translation of LXR-based therapies.

## 8. Future Directions

A major unresolved question in the field is whether LXR directly regulates structural myelin genes or whether increased myelin marker expression is primarily an indirect consequence of improved lipid homeostasis, reduced inflammation, and enhanced oligodendrocyte maturation. Direct LXR regulation is well established for canonical lipid-handling genes such as ABCA1, ABCG1, ApoE, and related cholesterol transport programs. In contrast, promoter-level evidence for direct LXR binding to classical myelin genes such as MBP, PLP1, MAG, MOG, CNP, MYRF, and SOX10 remains less definitive and should be interpreted with caution. Future studies should combine ChIP-seq, LXRE promoter mapping, reporter assays, CRISPR-based promoter disruption, and oligodendrocyte-specific transcriptomics to distinguish direct transcriptional targets from downstream myelin-associated outputs.

A second limitation is pharmacokinetic translation. Many LXR agonists were developed as systemic metabolic probes, not CNS-directed remyelinating therapies. As a result, brain penetration, plasma ratios, half-life, receptor occupancy, and CNS target engagement are not consistently reported across compounds. This creates uncertainty when interpreting preclinical efficacy, especially when peripheral LXR activation may dominate the pharmacodynamic response. Future studies should establish consistent PK/PD testing methods, including measuring how much drug reaches the brain compared with the blood, how long it remains active, and whether it effectively activates LXR signaling in brain cells. At the same time, safety markers such as liver fat accumulation, increased triglycerides, and activation of fat-producing genes should be monitored to ensure that beneficial brain effects are achieved without harmful metabolic side effects. Improving brain delivery while reducing effects on peripheral organs will be important for future LXR therapies. Approaches such as intranasal delivery may improve drug access to the brain, while nanoparticle-based systems could enhance drug stability and allow more targeted delivery. CNS-restricted prodrugs represent another potential strategy by activating the drug mainly within brain tissue while reducing exposure to the liver.

Collectively, the evidence supports a shift in the conceptual framework for LXR biology in neurodegenerative disease from the regulation of cholesterol metabolism to the therapeutic restoration of oligodendrocyte lipid homeostasis and myelin integrity. Overall, LXR signaling remains an important therapeutic target because it sits at the intersection of cholesterol efflux, ApoE lipidation, inflammatory resolution, glial repair, and oligodendrocyte-associated myelin maintenance. Although broad systemic LXR activation can produce dose-limiting effects such as hepatic lipogenesis, hypertriglyceridemia, fatty liver, and immune-related concerns, these limitations do not diminish the biological importance of the pathway. Instead, they highlight the need for more refined therapeutic strategies that preserve CNS-relevant LXR activity while minimizing peripheral toxicity. Approaches such as CNS-restricted delivery, LXRβ-biased agonism, partial agonism, prodrug design, and indirect modulation of endogenous oxysterol pathways such as CYP46A1/24S-OHC may allow future studies to harness the beneficial effects of LXR signaling more selectively. Therefore, LXR should be viewed as a highly relevant and mechanistically valuable pathway for promoting lipid homeostasis, reducing neuroinflammation, and supporting a CNS environment favorable for myelin repair.

## 9. Conclusions

In conclusion, LXR signaling represents a mechanistic link between brain cholesterol homeostasis, glial lipid handling, neuroinflammation, and myelin integrity. The 24S-OHC-LXR-ABCA1/ABCG1-ApoE axis provides a biologically plausible pathway through which neuronal cholesterol turnover can influence oligodendrocyte function, myelin maintenance, and remyelination capacity. However, therapeutic success will depend on overcoming major barriers, including poor CNS delivery, systemic LXRα-mediated lipogenesis, limited isoform selectivity, and insufficient disease-relevant validation. Next-generation LXRβ-biased, CNS-restricted, or CYP46A1/24S-OHC-modulating strategies may offer a more precise way to harness LXR signaling for myelin repair. Overall, targeting this pathway should be viewed not as a standalone cure for neurodegeneration, but as a rational strategy to restore lipid balance, improve glial support, and promote a CNS environment more permissive to myelin repair.

## Figures and Tables

**Table 1:** LXR molecule-based Strategies for Restoring Lipid Homeostasis and Remyelination.

Compound	LXR Activity	Myelin-Relevant Outcomes	CNS Exposure	Major Safety Findings	References
24S-hydroxycholesterol (24S-OHC)	Endogenous LXR ligand; activates LXRα/β	Activates LXR-dependent cholesterol transport pathways; supports lipid handling required for myelin maintenance	Endogenous CNS-derived oxysterol; physiological LXR activation	Dysregulated oxysterol balance may contribute to pathology	[90]
T0901317	Synthetic pan-LXR agonist (LXRα/β full agonist)	Increases cholesterol efflux genes, ApoE lipidation, and remyelination-associated responses	CNS activity reported, but systemic exposure predominates	Hepatic lipogenesis, hypertriglyceridemia, steatosis	[8,57]
GW3965	Synthetic LXRα/β agonist	Enhances ABCA1/ABCG1/ApoE pathways and lipid redistribution	CNS target engagement reported in preclinical studies	Hepatic lipid accumulation limits translation	[8,57]
DMHCA	Partial LXR modulator	Promotes cholesterol transport and remyelination while reducing lipogenic effects	CNS effects reported; exposure varies by study	Reduced but not eliminated metabolic toxicity	[91]
LXR-623	LXRβ-biased/selective modulator	Enhances LXR target gene activation and lipid homeostasis	CNS penetration demonstrated	Reduced peripheral toxicity compared with pan agonists	[92]
AU403	LXRβ/PPARδ dual modulator	Enhances lipid transport, cholesterol turnover, ApoE-mediated lipid handling, and myelin-supportive pathways	CNS target engagement requires further characterization	Designed to reduce metabolic liabilities associated with LXRα activation	[42]

## Data Availability

No new data were created or analyzed in this study. Data sharing is not applicable to this article.

## Abbreviations

24S-OHC	24S-Hydroxycholesterol
25-OHC	25-Hydroxycholesterol
27-OHC	27-Hydroxycholesterol
ABCA1	ATP-Binding Cassette Transporter A1
ABCG1	ATP-Binding Cassette Transporter G1
AD	Alzheimer’s Disease
ALS	Amyotrophic Lateral Sclerosis
ApoE/APOE	Apolipoprotein E
Aβ	Amyloid Beta
BBB	Blood–Brain Barrier
C9ORF72	Chromosome 9 Open Reading Frame 72
CH25H	Cholesterol 25-Hydroxylase
CNP	2′,3′-Cyclic Nucleotide 3′-Phosphodiesterase
CNPase	2′,3′-Cyclic Nucleotide 3′-Phosphodiesterase
CNS	Central Nervous System
CYP27A1	Cytochrome P450 Family 27 Subfamily A Member 1
CYP46A1	Cytochrome P450 Family 46 Subfamily A Member 1
DBD	DNA-Binding Domain
DHCR24	24-Dehydrocholesterol Reductase
DNA	Deoxyribonucleic Acid
EAE	Experimental Autoimmune Encephalomyelitis
FASN	Fatty Acid Synthase
FDA	Food and Drug Administration
FTD	Frontotemporal Dementia
FXR	Farnesoid X Receptor
GRN	Granulin/Progranulin Gene
HDAC3	Histone Deacetylase 3
HDL	High-Density Lipoprotein
HMGCR	3-Hydroxy-3-Methylglutaryl-CoA Reductase
HMGCS1	3-Hydroxy-3-Methylglutaryl-CoA Synthase 1
IL-1β	Interleukin-1 Beta
IL-6	Interleukin-6
iPSC	Induced Pluripotent Stem Cell
LBD	Ligand-Binding Domain
LDL	Low-Density Lipoprotein
LDLR	Low-Density Lipoprotein Receptor
LRP1	Low-Density Lipoprotein Receptor-Related Protein 1
LXR	Liver X Receptor
LXRα	Liver X Receptor Alpha
LXRβ	Liver X Receptor Beta
LXRE	Liver X Receptor Response Element
MAG	Myelin-Associated Glycoprotein
MAPT	Microtubule-Associated Protein Tau
MBP	Myelin Basic Protein
MCT1	Monocarboxylate Transporter 1
MOG	Myelin Oligodendrocyte Glycoprotein
MS	Multiple Sclerosis
MYRF	Myelin Regulatory Factor
NCoR	Nuclear Receptor Corepressor
NKX2.2	NK2 Homeobox 2
NPC1	Niemann–Pick Disease Type C1 Protein
NPC2	Niemann–Pick Disease Type C2 Protein
NR	Nuclear Receptor
NR1H2	Nuclear Receptor Subfamily 1 Group H Member 2 (LXRβ)
NR1H3	Nuclear Receptor Subfamily 1 Group H Member 3 (LXRα)
OLIG1	Oligodendrocyte Transcription Factor 1
OLIG2	Oligodendrocyte Transcription Factor 2
OPC	Oligodendrocyte Precursor Cell
PDGFRα	Platelet-Derived Growth Factor Receptor Alpha
PI3K	Phosphoinositide 3-Kinase
PLP/PLP1	Proteolipid Protein/Proteolipid Protein 1
PPAR	Peroxisome Proliferator-Activated Receptor
PPARγ	Peroxisome Proliferator-Activated Receptor Gamma
PPARδ	Peroxisome Proliferator-Activated Receptor Delta
PRMT5	Protein Arginine Methyltransferase 5
ROS	Reactive Oxygen Species
RXR	Retinoid X Receptor
SMRT	Silencing Mediator for Retinoid and Thyroid Hormone Receptors
SOD1	Superoxide Dismutase 1
SOX10	SRY-Box Transcription Factor 10
SREBF2	Sterol Regulatory Element-Binding Transcription Factor 2
SREBP	Sterol Regulatory Element-Binding Protein
SREBP-1c	Sterol Regulatory Element-Binding Protein 1c
SREBP2	Sterol Regulatory Element-Binding Protein 2
TDP-43	TAR DNA-Binding Protein 43
TMEV	Theiler’s Murine Encephalomyelitis Virus
TMEM106B	Transmembrane Protein 106B
TNF-α	Tumor Necrosis Factor Alpha
TREM2	Triggering Receptor Expressed on Myeloid Cells 2
VLDL	Very-Low-Density Lipoprotein
